# Supplementary material for: Genomic Landscape of Branchio-Oto-Renal Syndrome through Whole-Genome Sequencing: A Single Rare Disease Center Experience in South Korea
Source: Int J Mol Sci. 2024 Jul 26;25(15):8149. doi: 10.3390/ijms25158149 (PMC11311636; doi:10.3390/ijms25158149)
Supplement: Supplementary file 1 [file ijms-25-08149-s001.zip › ijms-3113068-supplementary.pdf]

BOR05-1452

A

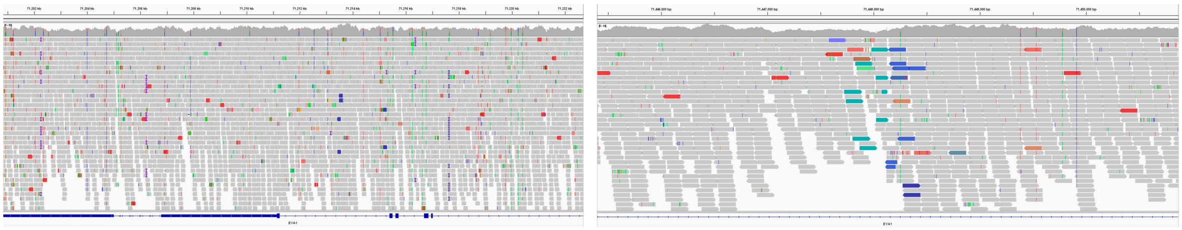

B

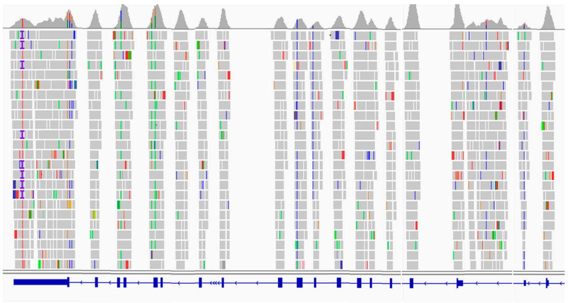

BOR02-1087

C

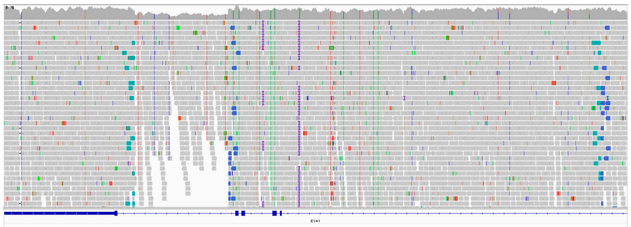

D

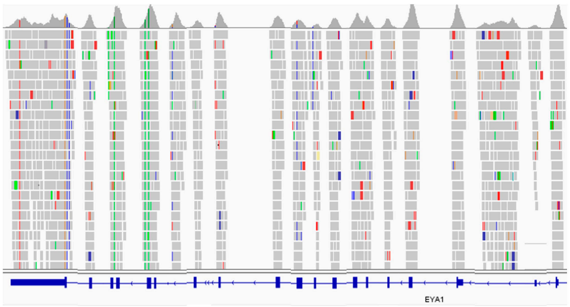

Figure S1: Comparing IGV (Integrative Genomic Viewer) diagram of WGS and WES. A. IGV diagram based on WGS of BOR05-1452. B. IGV diagram based on WES of BOR05-1452. C. IGV diagram based on WGS of BOR02-1087. D. IGV diagram based on WES of BOR02-1087.
